# Conductive hearing loss from ossicular compression by aberrant vessel

**DOI:** 10.1093/jscr/rjaf711

**Published:** 2025-09-06

**Authors:** Homood M Almutairi, Noura F Alanazi, Abdulrahman Fahad Alboqamie, Lulwah Alalayat

**Affiliations:** Department of Otolaryngology - Head and Neck Surgery, Prince Sultan Military Medical City, Makkah Al Mukarramah Branch Road, As Sulimaniyah District, Riyadh 11159, Saudi Arabia; College of Medicine, King Saud bin Abdulaziz University for Health Sciences, Prince Muteb bin Abdullah bin Abdulaziz Road, Al Rimayah District, Riyadh 11426, Saudi Arabia; King Abdullah International Medical Research Center (KAIMRC), Prince Muteb bin Abdullah bin Abdulaziz Road, Al Rimayah District, Riyadh 11481, Saudi Arabia; Neuroradiology Department, Prince Sultan Military Medical City, Makkah Al Mukarramah Branch Road, As Sulimaniyah District, Riyadh 11159, Saudi Arabia; Department of Otolaryngology - Head and Neck Surgery, Prince Sultan Military Medical City, Makkah Al Mukarramah Branch Road, As Sulimaniyah District, Riyadh 11159, Saudi Arabia

**Keywords:** conductive hearing loss, vascular ossicular compression, middle ear vascular anomaly

## Abstract

Middle ear vascular anomalies are an uncommon and frequently overlooked cause of conductive hearing loss (CHL). Their clinical presentation can mimic more prevalent conditions, making diagnosis and management challenging. Without appropriate imaging and evaluation, such cases may lead to unnecessary and potentially harmful interventions. We report the case of a 31-year-old woman with chronic right-sided CHL due to a vascular anomaly compressing the ossicles. This case underscores the critical role of high-resolution imaging in diagnosing rare etiologies of CHL and guiding safe, informed management.

## Introduction

Conductive hearing loss (CHL) encompasses a wide range of pathologies affecting all age groups. It most commonly arises from abnormalities in the external ear, tympanic membrane, or ossicular chain, including the stapes footplate [1]. Common causes include otosclerosis, middle ear effusion, and ossicular chain disruption.

Vascular anomalies implicated in middle ear pathology include persistent stapedial arteries and, high-riding jugular bulbs, and aberrant internal carotid artery (ICA) each of which can mimic or contribute to conductive hearing loss [2–4]. Although they are a rare cause, these anomalies can lead to CHL by directly compressing the ossicular chain and interfering with normal sound transmission. Failure to recognize such anomalies may result in unnecessary surgical exploration or interventions, potentially leading to serious complications.

While clinical history and physical examination remain essential diagnostic tools, high-resolution computed tomography (CT) of the temporal bone is invaluable in identifying atypical causes of CHL, including vascular or middle ear abnormalities. However, such vascular compressions are rarely reported and can complicate the clinical presentation.

To our knowledge, no similar case has been reported in the current literature. Thus, in this case report, we shed light on an unusual case of right-sided CHL in an adult female, caused by vascular compression of the ossicles. It highlights the importance of considering middle ear pathology and vascular anomalies in the differential diagnosis of unexplained conductive hearing loss.

## Case presentation

A 31-year-old female with a known history of hypothyroidism presented to our institution’s otorhinolaryngology clinic with a longstanding complaint of decreased hearing in the right ear. She denied any associated symptoms, such as otorrhea, recurrent ear infections, tinnitus, or vertigo. Otoscopic examination revealed intact tympanic membranes bilaterally, with no visible middle ear abnormalities. Endoscopic examination was also unremarkable.

**Figure 1:**
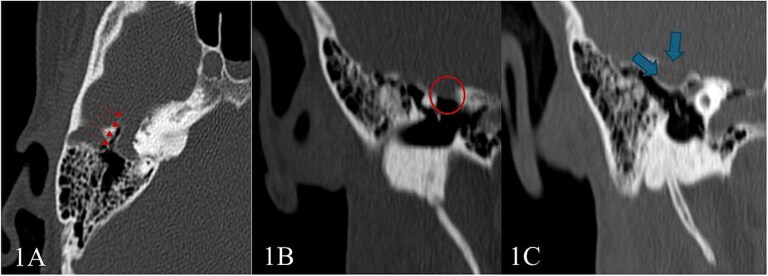
High-resolution CT scan of the temporal bone shows: (A) Tubular lucency noted lateral to superior semicircular canal with associated dehiscence. (B and C) Scalloping and protrusion of right tegmen tympani into epitympanum with a marked narrowing of epitympanum recess. Additionally, there is a lateralization of ossicular chain and apparent subluxation of incudomalleolar joint.

Pure tone audiometry demonstrated mild low-frequency conductive hearing loss in the right ear, while hearing thresholds were within normal limits in the left ear. Given these findings, a high-resolution CT scan of the temporal bones was performed, revealed a tubular lucency lateral to superior semicircular canal with associated dehiscence. Additionally, lateralization of the ossicular chain and apparent subluxation of incudomalleolar joint were observed (Fig. 1). This was associated with an undulating contour suggestive of a possible intracranial component, raising suspicion for a small meningocele or cephalocele—warranting further evaluation with MRI.

**Figure 2:**
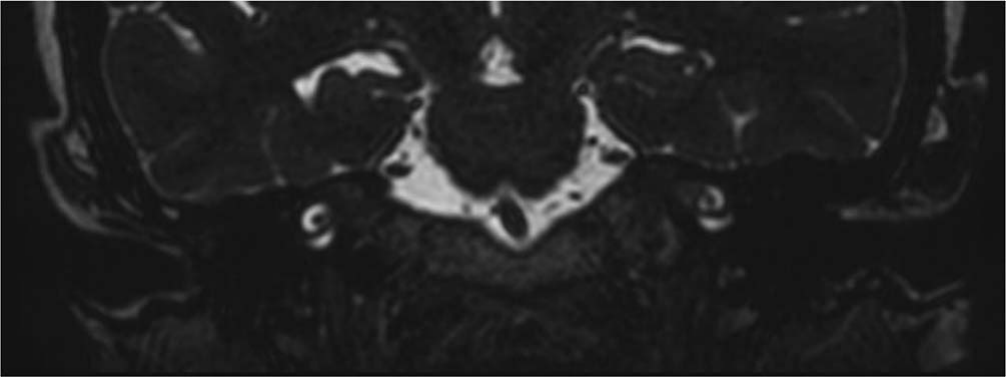
Coronal T2 FIESTA confirm the absence of encephalocele.

**Figure 3:**
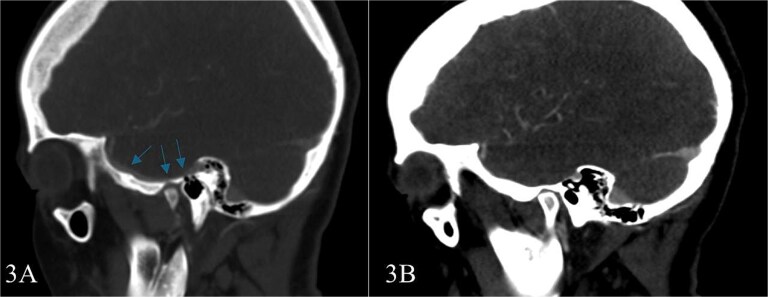
Consecutive images from late arterial phase CT scan reveals; the vascular nature of aforementioned observation which drain into dural venous sinus.

Subsequent MRI showed no evidence of meningocele or cephalocele, confirming an unremarkable intracranial evaluation (Fig. 2).To further evaluate potential vascular anomalies, a diagnosis of possible vascular compression over the ossicles was considered. Consecutive images from late arterial phase CT scan revealed the vascular nature of observation which drain into dural venous sinus (Fig. 3). A 3D cerebral angiogram was performed and confirmed the presence of a venous protrusion in the middle ear; however, the venous protrusion was not accessible for endovascular ligation due to its anatomical location and limited accessibility, making the risk of failed intervention high. Multidisciplinary discussions were held to consider management options, including middle ear exploration with attempted dissection of the venous anomaly from the ossicles. An alternative approach discussed was attic bypass via incus dislocation followed by ossiculoplasty. Given the patient’s stable condition and minimal impact on quality of life the patient preferred to pursue conservative management with hearing amplification and was scheduled for ongoing follow-up.

## Discussion

Vascular anomalies of the middle ear are uncommon but important causes of CHL. Their diagnosis can be challenging, particularly when otoscopic examination and middle ear evaluation appear normal. In such situations, high-resolution imaging plays a pivotal role in revealing underlying pathology. Our case emphasizes the need for a multidisciplinary approach—often involving otology, radiology, and occasionally neurosurgery to ensure accurate diagnosis and safe management. Table 1 summarizes the vascular anomalies reported in the literature, their clinical presentations, and the management strategies.

**Table 1:** Summary of reported middle ear vascular anomalies

Title	Vascular Anomaly	Patient	Presentation	Management
Eryilmaz *et al*. [4]	Aberrant internal carotid artery	48-year-old female	Conductive hearing loss and retro-tympanic blue-reddish mass	Conservative management
Endo *et al*. [6]	Aberrant internal carotid artery	37-year-old female	Pulsatile tinnitus, conductive hearing loss, and retro-tympanic white mass	Conservative management; regular follow-up
Schaumberg *et al*. [7]	Aberrant internal carotid artery	58-year-old female	pulse-synchronous tinnitus	Conservative management
Lapayowker *et al*. [8]	Aberrant internal carotid artery	50-year-old female	Fullness and retro-tympanic pulsatile mass	Surgical management
		22-year-old female	Asymptematic retro-tympanic pulsatile mass	Conservative management
		11-year-old female	Intermittent earache, buzzing	Surgical management
Silbergleit *et al*. [10]	Persistent stapedial artery	4-year-old female	Otorrhagia during attempted myringotomy	Conservative management
		6-year-old male	Otorrhagia during	
		6-year-old female	Otorrhagia during grommet insertion myringotomy, red mass	
		5-year-old female	Bleeding during tympanostomy	
		5-year-old male	Massive bleeding during myringotomy	
Jones *et al*. [12]	Persistent stapedial artery	25-year-old male	PSA identified intraoperatively	Successful CI with intraoperative adaptation
Libeert *et al*. [13]	Persistent stapedial artery	25-year-old male	Conductive hearing loss, “plop” sound	Conservative management; follow-up
Santiago *et al*. [3]	High riding jugular bulb and attic cholesteatoma	36-year-old female	Pulsatile tinnitus, fullness, and conductive hearing loss	Endoscopic intervention of the attic cholesteatoma
Totten [14]	High riding jugular bulb	13-year-old male	Persistent CHL post‑tympanostomy	Conservative management; follow-up

One of the most well-documented anomalies is the aberrant ICA. Although rare, with an estimated prevalence of ~1% [5], its proximity to the ossicular chain can lead to CHL, pulsatile tinnitus, or vertigo. Eryilmaz *et al*. reported an adult female with unilateral CHL in whom otoscopy revealed a bluish-red retrotympanic mass. High-resolution CT and MR angiography confirmed an aberrant ICA protruding into the middle ear [4]. Similarly, Endo *et al*. reported a patient with pulsatile tinnitus and CHL due to an aberrant ICA presenting as a retrotympanic mass, again emphasizing the importance of thorough imaging [6]. Similarly, Endo *et al*. [5] described a patient with pulsatile tinnitus and CHL where the retrotympanic mass was ultimately diagnosed as an aberrant ICA following detailed imaging. Schaumberg *et al*. presented the case of a 58-year-old woman with pulse-synchronous tinnitus, where temporal bone CT demonstrated an aberrant ICA traversing the middle ear with a narrow carotid canal [7]. Lapayowker *et al*. added three more cases: one treated surgically after recurrent vertigo and failed conservative measures, one managed conservatively after angiographic confirmation, and the third case involved intraoperative clipping of the vessel to control bleeding, with no further resection attempted [8]. These reports underline both the variable clinical presentation of aberrant ICA and the importance of individualized management strategies.

Another significant vascular anomaly is the persistent stapedial artery (PSA), resulting from failure of embryologic vascular structures regression. PSA typically presents as a pulsatile retrotympanic mass, pulsatile tinnitus, and CHL. Hunter *et al*. provided a comprehensive review of PSA imaging features, highlighting the role of high-resolution CT and MRI in identifying this anomaly and avoiding misdiagnosis or surgical complications [9]. Silbergleit *et al*. described five PSA cases—one symptomatic with pulsatile tinnitus, the others incidental findings during evaluations for chronic otitis media or CHL. Imaging typically revealed a non-erosive tubular structure traversing the promontory and connecting to the internal carotid canal. One case coexisted with an aberrant ICA, greatly increasing operative risk [10].

The high-riding jugular bulb is another vascular variant that can contribute to CHL by physically encroaching upon the ossicular chain or middle ear space. Santiago and Jorge reported an unusual case involving concurrent attic cholesteatoma and high-riding jugular bulb in the same ear. In this case, the jugular bulb mimicked ossicular erosion often seen with cholesteatoma, creating a diagnostic challenge [3]. In the pediatric population, Koo *et al*. described a 9-year-old boy presenting with a red-to-purplish retrotympanic mass and mild right-sided CHL. Although initially suspected to be a hypervascular tumor such as glomus tympanicum, temporal bone CT revealed a dehiscent high-riding jugular bulb protruding into the middle ear cavity, avoiding unnecessary surgical intervention [11].

Vascular anomalies in the middle ear can interfere with sound conduction and pose serious intraoperative risks. Accurate preoperative identification through high-resolution CT and magnetic resonance angiography is essential for appropriate surgical planning. The present case reinforces the need to consider vascular anomalies in the differential diagnosis of unexplained CHL, particularly when tympanic membrane appearance and middle ear examination are unremarkable. Moreover, it underscores the vital role of imaging in guiding both diagnostic and therapeutic strategies.

## Conclusion

This study highlights the critical role of high-resolution imaging in the evaluation of patients with unexplained conductive hearing loss. When clinical assessment fails to identify an obvious cause, radiologic evaluation becomes essential—not only for diagnosis but also for detecting rare vascular anomalies that, if unrecognized, may lead to serious intraoperative complications. Prompt and accurate imaging is therefore vital for establishing the correct diagnosis and guiding safe and appropriate management.
